# Targeting Health and Functioning Barriers to Social Connection to Reduce Suicide Risk

**DOI:** 10.1093/geroni/igaf122.1214

**Published:** 2025-12-31

**Authors:** Kimberly Van Orden

**Affiliations:** University of Rochester Medical Center, Rochester, New York, United States

## Abstract

Changes in health and functioning can introduce barriers to social engagement for older adults. Social disconnection—low social engagement, isolation, and loneliness—is associated with suicide ideation (SI), attempts, and deaths in later life. However, it is not known if promoting social connection is an effective strategy to prevent suicide in later life because studies have not directly tested this mechanism. This presentation will describe results from a clinical trial testing a brief behavioral intervention to increase social engagement among older adults experiencing clinically significant loneliness. Severity of SI via 10 days of ecological momentary assessment (EMA) at baseline and 3-month follow-up will be presented. The Geriatric Suicide Ideation Scale, Screening Version was used in EMA (4 items rated 1-3; possible scores 4-12). Currently, 29 out of 30 planned subjects have been assigned to the study intervention and completed the EMA protocol at baseline; 13 subjects completed follow-up EMA. The sample has an average age of 72.26 years (range 60-96); 5 subjects are male, 19 female, and 1 intersex. Just under half the sample (48%) reported suicide ideation (lifetime) at baseline. SI severity (GSIS) was 6.71 (std 1.71) via EMA at baseline and decreased to 6.40 (std 2.13) at follow-up (p = 0.001). Presentation results will include the full sample and effect sizes to illustrate magnitude of effects. Results indicate reliable reductions in suicide ideation severity after 10 sessions of one-on-one social engagement coaching to address functioning and health-related barriers, consistent with potential benefits of this approach for late-life suicide prevention.

